# *Post*operative but not *pre*operative depression is associated with cognitive impairment after cardiac surgery: exploratory analysis of data from a randomized trial

**DOI:** 10.1186/s12871-022-01672-y

**Published:** 2022-05-23

**Authors:** Choy Lewis, Mehmet E. Dokucu, Charles H. Brown, Lauren Balmert, Nina Srdanovic, Ashwin Shaan Madhan, Sahej Singh Samra, John Csernansky, Jordan Grafman, Charles W. Hogue

**Affiliations:** 1 Department of Anesthesiology, The Bluhm Cardiovascular Institute, Northwestern University Feinberg School of Medicine, 251 East Huron St, Feinberg 5-704, Chicago, IL 60611 USA; 2 Department of Psychiatry, Northwestern University Feinberg School of Medicine, Chicago, IL USA; 3 Department of Anesthesiology & Critical Care Medicine, The Johns Hopkins University School of Medicine, Baltimore, MD USA; 4 Department of Preventative Medicine, Northwestern University Feinberg School of Medicine, Chicago, IL USA; 5 Medical Student, Northwestern University Feinberg School of Medicine, Chicago, IL USA; 6 Department of Physical Medicine & Rehabilitation, Neurology, Cognitive Neurology and Alzheimer’s Center, Department of Psychiatry, Northwestern University Feinberg School of Medicine, Weinberg College of Arts and Sciences, Northwestern University, Chicago, IL USA

**Keywords:** Cardiac surgery, Depression, Neurocognitive dysfunction, Quality of life

## Abstract

**Background:**

In this study we hypothesize that depression is associated with perioperative neurocognitive dysfunction and altered quality of life one month after surgery.

**Methods:**

Data were obtained as part of a study evaluating cerebral autoregulation monitoring for targeting arterial pressure during cardiopulmonary bypass. Neuropsychological testing was performed before surgery and one month postoperatively. Testing included the Beck Depression Inventory, a depression symptoms questionnaire (0–63 scale), as well as anxiety and quality of life assessments. Depression was defined as a Beck Depression Inventory score > 13.

**Results:**

Beck Depression data were available from 320 patients of whom cognitive domain endpoints were available from 88–98% at baseline and 69–79% after surgery. This range in end-points data was due to variability in the availability of each neuropsychological test results between patients. Depression was present in 50 (15.6%) patients before surgery and in 43 (13.4%) after surgery. Baseline depression was not associated with postoperative domain-specific neurocognitive function compared with non-depressed patients. Those with depression one month after surgery, though, had poorer performance on tests of attention (*p* = 0.017), memory (*p* = 0.049), verbal fluency (*p* = 0.010), processing speed (*p* = 0.017), and fine motor speed (*p* = 0.014). Postoperative neurocognitive dysfunction as a composite outcome occurred in 33.3% versus 14.5% of patients with and without postoperative depression (*p* = 0.040). Baseline depression was associated with higher anxiety and lower self-ratings on several quality of life domains, these measures were generally more adversely affected by depression one month after surgery.

**Conclusions:**

The results of this exploratory analysis suggests that preoperative depression is not associated with perioperative neurocognitive dysfunction, but depression after cardiac surgery may be associated with impairment in in several cognitive domains, a higher frequency of the composite neurocognitive outcome, and altered quality of life.

**Trial Registration:**

www.clinicaltrials.gov, NCT00981474 (parent study).

**Supplementary Information:**

The online version contains supplementary material available at 10.1186/s12871-022-01672-y.

## Background

Approximately 15% of adults in the United States suffer from a major depressive disorder leading to reduced work productivity, compromised health, and reduced survival [1, 2]. The prevalence of depression is reported to be even higher in patients with cardiac diseases including those undergoing cardiac surgery where it is reported in up to 40% of patients [3–6]. Symptoms of depression at the time of surgery is associated with risk for postoperative complications, reduced functional recovery, impaired quality of life, and mortality [3, 4, 6–8]. Science Advisory Committee recommendations from the American Heart Association, in fact, recommend screening for depression in patients with coronary artery disease including those undergoing surgery [9]. Nonetheless, a mechanistic explanation for why pre-existing depression contributes to adverse postoperative outcomes is unclear [10].

Perioperative neurocognitive disorders contribute to patient morbidity after cardiac surgery [11]. These disorders are manifest early after surgery as delirium or in the weeks to months later as postoperative neurocognitive dysfunction [12]. We have found that a history of depression is two to three times more common in patients developing postoperative delirium than in those without delirium [13, 14]. Further, we have found an association between depression within a year of cardiac surgery and perceived cognitive difficulties [4]. Less is known, however, on the role that depression plays in neurocognitive dysfunction one month after surgery. Understanding if this link is present might provide insight into whether attenuating depressive symptoms is a plausible strategy for improving neurocognitive recovery from cardiac surgery. The aim of this study was to evaluate whether there is a relationship between depression either before or after cardiac surgery and postoperative neurocognitive dysfunction. Impairment of individual cognitive domains that comprise a composite neurocognitive outcome identifies risk for postoperative delirium in depressed patients after non-cardiac surgery even in the absence of global cognitive impairment [15]. Thus, we focus on an evaluation of domain-specific cognitive performance. We hypothesize that preoperative and/or postoperative depression is associated with reduction from baseline in domain-specific cognitive function one month after surgery compared with patients not having depression.

## Methods

This study is an exploratory analysis of data collected from a prospective randomized clinical investigation of 460 patients evaluating whether targeting mean arterial pressure during cardiopulmonary bypass to be above the lower limit of cerebral autoregulation improves neurological outcomes compared with usual care [16]. The results of that study have been reported in accordance with guidelines of Consolidated Standards of Reporting Trials. The parent study was approved by the Institutional Review Boards of The Johns Hopkins Medical Institutions, Baltimore, MD and Northwestern University, Chicago, IL. Patients provided written informed consent to participate in the study.

### Patients

Patients aged ≥ 55 years with planned CABG with or without cardiac valve surgery, valve surgery, or ascending aortic surgery were eligible for enrollment in the parent trial if they were at risk for neurological complications based on history of stroke, and/or carotid bruit, and/or hypertension, and/or diabetes Patients were excluded if there was a contraindication to MRI; if the estimated glomerular filtration rate was ≤ 60 mL/min or they were on current renal dialysis; if they were receiving emergency surgery; inability to attend outpatient visits; or if they had visual impairment or if they were unable to speak and read English. The patients were enrolled between October 2, 2009 and January 10, 2019 at The Johns Hopkins Hospital, Baltimore, MD and Northwestern Memorial Hospital, Chicago, IL. The study was approved by the Institutional Review Board Committees of The Johns Hopkins Medical Institutes (Baltimore, MD) and Northwestern University Feinberg School of Medicine (Chicago, IL). Written informed consent was obtained from all patients.

### Intraoperative management

The patients received a benzodiazepine, propofol, fentanyl, isoflurane, and a skeletal muscle relaxant for anesthesia. Cerebral blood flow autoregulation monitoring was performed after anesthesia induction using transcranial Doppler (Compumedics DWL, El Paso, TX) method as we have described [16, 17]. Non-pulsatile cardiopulmonary bypass was conducted using a membrane oxygenator, flow of 2.0 to 2.4 L/min/m^2^, and using α-stat pH management. Arterial pH and PaCO_2_ were maintained within a normal range by continuous in-line monitoring calibrated with arterial blood gases. Patients were randomized after anesthesia induction to have their mean arterial blood pressure targets during cardiopulmonary bypass to be above the lower limit of autoregulation or based on usual institutional practice [16]. Blood pressure was raised with phenylephrine (100 mcg) and by reducing isoflurane concentrations to 0.7% after first ensuring target CPB flow. Patients and assessors were blinded to randomization group.

### Neuropsychological and neurological evaluations

Neuropsychological testing was performed one to two days before surgery [16, 18]. This testing was repeated one month (four to six weeks) after surgery during the patient’s postoperative clinic visit with their surgeon. The cognitive testing battery evaluated domains reported to be adversely affected by cardiac surgery [19]. The battery consisted of the Rey Auditory Verbal Learning Test; Rey Complex Figure Test; Symbol Digit Modalities Test; Trail Making A and B; Controlled Oral Word Association Test; and the Grooved Pegboard Test as we have previously described [16, 18, 20]. The tests were grouped into the following cognitive domains: *attention* (Rey Auditory Verbal Learning Test I correct); *memory* (Rey Auditory Verbal Learning Test V correct, Rey Auditory Verbal Learning Test IX correct); *visuoconstruction* (Rey Complex Figure Test copy trial score); *verbal fluency* (Controlled Oral Word Association Test letters F, A, S); *processing speed* (Symbol Digit Modalities Test correct, Trail Making Test A); *executive function* (Trail Making Test B); and *fine motor speed* (Grooved Pegboard, dominant and non-dominant hand) [14, 18]. The Beck Depression Inventory was administered to assess depressive symptoms at each testing session [21]. This test is a 21-item questionnaire that assesses self-reported symptoms including such items as self-dislike, suicidal ideation, insomnia, and sadness. The score for each item ranges from 0–3 that are then summarized on a scale ranging from 0 to 63. Scores of 14–19, 20–28, 29–63 are considered to represent mild, moderate, and severe depression, respectively. A cut-off of > 13 was used as a dichotomous definition of depression. The patients were assessed at the same testing periods with the SF-36 short form of the RAND Medical Outcomes Study (MOS) health-related quality of life questionnaire [22]. This questionnaire is a multi-item scale that measures 8 health-related concepts: physical function, social function, physical role, emotional role, mental health, energy, pain, and general health perceptions. Anxiety was assessed using the Beck State-Trait Anxiety Inventory that assesses self-reported ratings of anxiety on a four-point Likert scale [23]. This inventory rates two types of anxiety: state anxiety or anxiety related to an event; and trait anxiety, or anxiety as a personal characteristic.

The patients were evaluated by their physicians and nurses daily for any acute mental status changes including abnormalities in arousal, memory, or cognition. A diagnosis of clinical delirium was entered into the medical record using International Classification of Diseases-10^th^ Revision (Codes F05 or G93.40) criteria.

### Outcomes

The primary outcomes were changes in cognitive function, defined as changes in Z-scores on each of the seven domains from baseline to one month postoperative time points. Secondary outcomes included the raw postoperative cognitive domain Z-scores, a composite postoperative neurocognitive dysfunction end-point, quality of life measurements, anxiety, and clinical complications (delirium, stroke, atrial fibrillation, intra-aortic balloon insertion, dialysis, multisystem organ failure, duration of ICU admission, duration of hospitalization, and death) [20]. Postoperative neurocognitive dysfunction was defined by a clinically meaningful decline in cognitive function based on a change in composite Z-score, comprising all individual cognitive tests. Specifically, individual Z-scores were combined into a global Z-score at each time-point calculated from the average of the individual cognitive tests. These were then re-normalized using the mean and standard deviation of the tests at baseline [20]. Timed tests were multiplied by ‘-1’ so that higher scores represented better performance. Prior studies have suggested that a decline in composite Z scores from baseline of 0.3 to 0.5 to be clinically important [20, 24]. A difference between pre- and post-test > 0.3 was considered as postoperative neurocognitive dysfunction [20].

### Primary exposure

The primary exposure was depressive symptomatology defined as a Beck Depression Inventory score > 13.

## Sample size

Insofar as the present study is a secondary analysis of a prior randomized clinical investigation, the sample size was set based on the parent study. There was no power calculation for the present study. The sample size for the parent study was based on detecting differences in the proportion of patients with a composite adverse neurologic outcome between the usual care and autoregulation groups.

## Data analysis

Descriptive statistics summarized all variables by preoperative depression. Fisher’s exact tests for categorical variables, two sample t-tests for normal continuous variables, and Wilcoxon rank sum tests for non-normal continuous variables, were used to compare distributions of baseline and intraoperative variables by depression status. A simple paired t-test assessed changes in Beck Depression Inventory from preoperative to the one month postoperative time points. Primary analyses utilized multivariable linear regression models to assess associations between preoperative depression and change in each domain Z-score from baseline to four to six week follow-up, adjusting for variables including: baseline domain Z-score, sex, age, education, parent study blood pressure treatment arm, diabetes, obesity, prior stroke, hypertension, atrial fibrillation, and COPD. A false discovery rate (FDR) correction was applied to correct for multiple testing [25]. This approach allows for controlling the expected proportion of tests which are falsely rejected. Due to missing data, sensitivity analyses were conducted for primary models using multiple imputation with 20 imputed datasets via multivariate imputation by chained equations, using the mice package in R [26]. Additional sensitivity analyses replicated models with the continuous Beck Depression Inventory score as the independent variable of interest. Secondary analyses considered similar models to assess associations between postoperative depression and the postoperative cognitive domain Z-scores, with similar adjustment for potential confounders. Generalized linear models with Poisson distributions and robust standard errors assessed associations between preoperative and postoperative depression, in turn, with the neurocognitive dysfunction indicator, adjusting for age, sex, education, treatment arm, diabetes, obesity, prior stroke, hypertension, atrial fibrillation, and COPD. Additional secondary analyses then considered the SF-36 scales and State Anxiety Inventory outcomes, measured at four to six weeks postoperative testing period, in similar models. Specifically, separate multivariable linear regression models were considered for preoperative depression and postoperative depression with each SF-36 scale and State-Trait Anxiety scale, with adjustment for age, sex, education, baseline score, treatment arm, diabetes, obesity, prior stroke, hypertension, atrial fibrillation, and COPD, unless otherwise specified in the results section. Due to violation of distributional assumptions, Physical Health Limits and Emotional Health Limits scales were dichotomized at 0 and considered in multivariable logistic regression models. Lastly, associations between depression and clinical complications were assessed using generalized linear models with Poisson distributions and robust standard errors for binary outcomes and multivariable linear regression models for continuous outcomes adjusted for treatment arm, sex, age, and education. A false discovery rate (FDR) correction was applied to correct for testing of the multiple cognitive domains, multiple quality of life scales, and clinical endpoints, within each set of analyses.

## Results

**Fig. 1:**
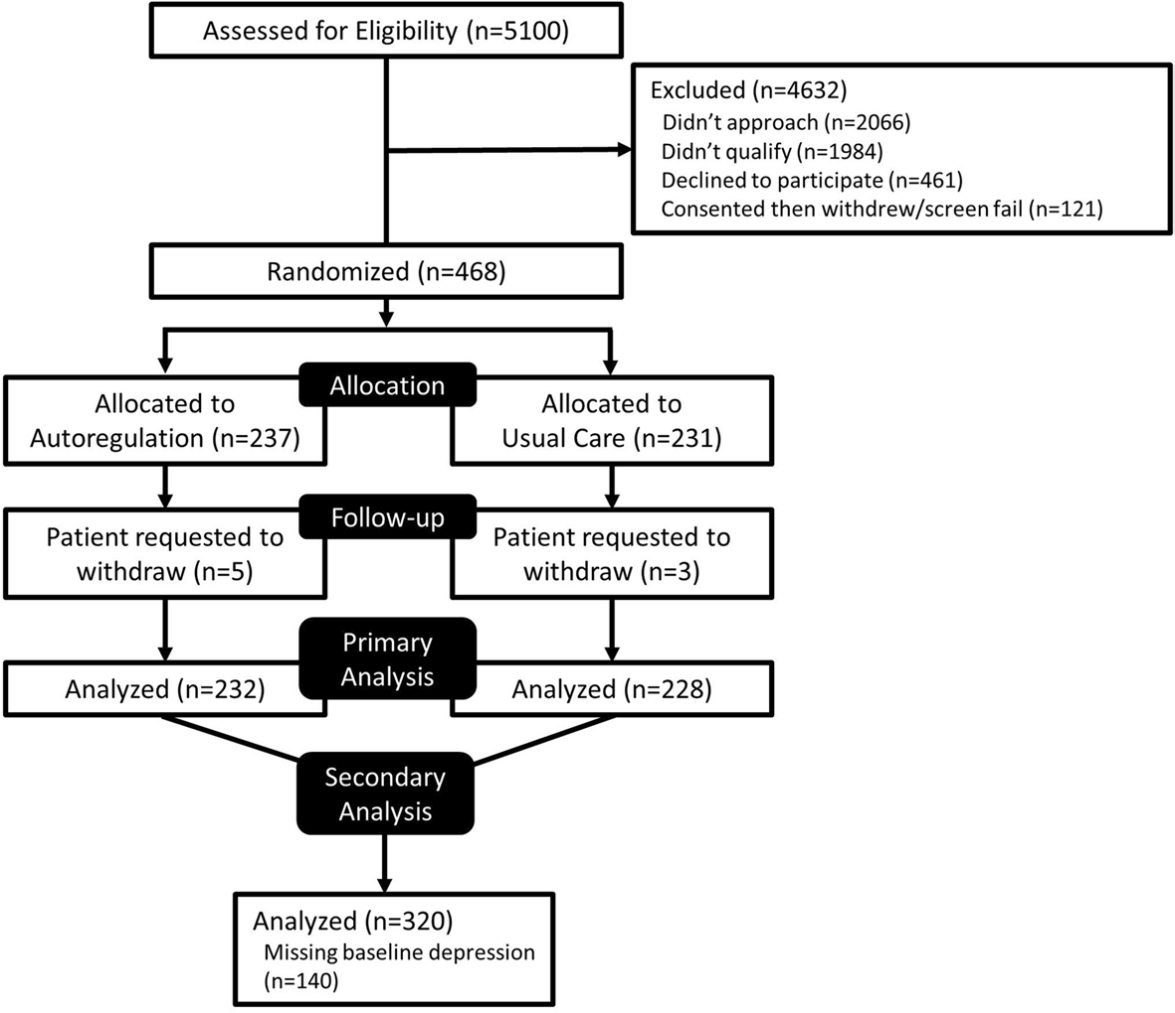
Study patient flow diagram

Preoperative Beck Depression Inventory data were available from 320 of the original 460 patients. Of these patients, complete cognitive domain endpoints were available for 88–98% at baseline and 69–79% at both baseline and the one-month follow-up testing session. This range in end-points data was due to variability in the availability of specific neuropsychological test results between patients. A patient flow diagram of the study is shown in Fig. 1. Depression was present at the time of surgery in 50 (15.6%) of the patients of whom 32 (10%) had mild depression, 8 (2.5%) moderate depression, and 10 (3.1%) severe depression. The characteristics of the entire cohort and for those with a Beck Depression Inventory score ≤ 13 and > 13 are listed in Table 1. Of note, there were no differences in patient characteristics for those with Beck Depression Inventory data compared to those with missing data (supplemental Table 1). Patients with preoperative depression were more likely to have had a history of prior stroke, chronic obstructive lung disease, and a lower level of attained education compared with patients without depression. There were no other differences between depressed and non-depressed patients for other variables including operative data or the cerebral autoregulation metrics during cardiopulmonary bypass. Depression was present in 43 (13.4%) of the 32 patients with Beck Depression Inventory data at the one month postoperative testing period with 34 (79.1%), 8 (18.6%), and 1 (2.3%) demonstrating mild, moderate, or severe depression, respectively. Of the 50 depressed patients *before* surgery, 34 had postoperative Beck Depression Inventory data, and 17 (50%) of those individuals remained depressed one month after surgery. Fourteen (6.6%) patients not demonstrating depression before surgery were now demonstrating depression at this latter time point.

**Table 1:** Patient demographic and operative characteristics for the entire cohort and for those with Beck Depression Inventory data (*n* = 320). The patients are categorized as having depression based on a Beck Depression Inventory score > 13 before surgery. The *p*-value listed is for comparison of each variable between patients without and with depression at the time of surgery

	**Total Cohort** ***n* = 460**	**No Depression** ***n* = 270**	**Depression** ***N* = 50**	***P*-Value**^**b**^	**Standardized Difference**
Age (mean (SD))	70.42 (7.66)	70.62 (7.54)	68.74 (8.45)	0.113	0.235
Female (%)^a^	129 (28.1)	71 (26.4)	18 (36.0)	0.172	0.208
Race (%)^a^				0.283	0.445
White	373 (81.3)	224 (83.3)	37 (74.0)		
African American	52 (11.3)	25 (9.3)	11 (22.0)		
Asian-Oriental	4 (0.9)	3 (1.1)	0 (0.0)		
Asian-Subcontinent	4 (0.9)	3 (1.1)	0 (0.0)		
Hispanic	5 (1.1)	3 (1.1)	0 (0.0)		
Multiple	2 (0.4)	1 0.4)	0 (0.0)		
Other	19 (4.1)	10 (3.7)	2 (4.0)		
BMI > 30 (%)	192 (41.7)	106 (39.3)	20 (40.0)	0.999	0.015
Diabetes (%)^a^	209 (46.1)	111 (41.3)	26 (54.2)	0.114	0.261
Hypertension (%)	420 (91.3)	240 (88.9)	49 (98.0)	0.064	0.375
Prior stroke (%)^a^	44 (10.0)	20 (7.7)	9 (19.1)	0.025	0.342
Prior myocardial infarction (%)^a^	139 (30.5)	75 (28.0)	15 (30.0)	0.864	0.044
Atrial fibrillation (%)^a^	102 (22.4)	62 (23.0)	9 (18.0)	0.579	0.125
Chronic obstructive pulmonary disease (%)^a^	52 (11.6)	28 (10.6)	11 (22.9)	0.030	0.333
Current tobacco use (%)^a^	46 (10.2)	25 (9.3)	6 (12.5)	0.441	0.102
Years of attained education (median [IQR])	14.00 [12.00, 17.00]	15.00 [12.00, 17.00]	12.00 [12.00, 16.75]	0.027	0.403
Anti-depressant drug usage (%)	62 (13.4)	33 (12.2)	10 (20)	0.172	0.213
Surgical procedure (%)				0.545	0.495
CABG	226 (49.1)	122 (45.2)	26 (52.0)		
CABG/AVR	61 (13.3)	36 (13.3)	9 (18.0)		
CABG/MVR	15 (3.3)	8 (3.0)	2 (4.0)		
CABG/AVR/MVR	89 (19.3)	55 (20.4)	11 (22.0)		
AVR	41 (8.9)	30 (11.1)	1 (2.0)		
MVR	6 (1.3)	5 (1.9)	0 (0.0)		
AVR/MVR	14 (3.0)	10 (3.7)	1 (2.0)		
Aortic root replacement	4 (0.9)	2 (0.7)	0 (0.0)		
CABG/Aortic Root Replacement	1 0.2)	0 (0.0)	0 (0.0)		
TVR	3 (0.7)	2 (0.7)	0 (0.0)		
**Surgical Parameters**					
Minutes of cardiopulmonary bypass (median [interquartile range])	104.0 [81.2, 140.7]	107.0 [82.0, 142.0]	103.0 [84.0, 132.0]	0.934	0.078
Minutes of aortic cross-clamping (median [interquartile range])	71.0 [54.0, 93.5]	73.0 [56.0, 94.0]	72.5 [55.0, 94.7]	0.959	0.001
Impaired cerebral autoregulation during cardiopulmonary bypass (%)	130 (28.7)	74 (27.9)	8 (16.7)	0.111	0.273
MAP (mmHg) at the lower limit of autoregulation (median [interquartile range])	65.0 [60.0, 75.0]	70.0 [60.0, 75.0]	65.0 [60.0, 70.0]	0.797	0.070
Area under the curve that MAP was ≤ the lower limit of cerebral autoregulation (median [interquartile range] mmHg x hr)^1^	6.75 [2.41, 14.78]	7.45 [2.66, 16.20]	4.60 [1.66, 10.24]	0.059	0.379

*CABG* coronary artery bypass graft surgery, *AVR* aortic valve replacement, *MVR* mitral valve repair or replacement, *TVR* tricuspid valve replacement;

^a^Missing excluded from summaries and denominators (age [*n* = 1], sex [*n* = 1]; race [*n* = 1]; diabetes [*n* = 3]; prior stroke [*n* = 12]; prior myocardial infarction [*n* = 2]; atrial fibrillation [*n* = 1]; chronic obstructive pulmonary disease [*n* = 9]; current tobacco use [*n* = 4]; years of attained education [*n* = 44]; minutes of cardiopulmonary bypass [*n* = 2]; impaired cerebral autoregulation [*n* = 7]; MAP [*n* = 5]; area under the curve [*n* = 5]);

^b^P-value from complete cases analysis via fisher’s exact for categorical variables, t-tests for normal continuous variables, and Wilcoxon rank sum tests for non-normal continuous variables

**Fig. 2:**
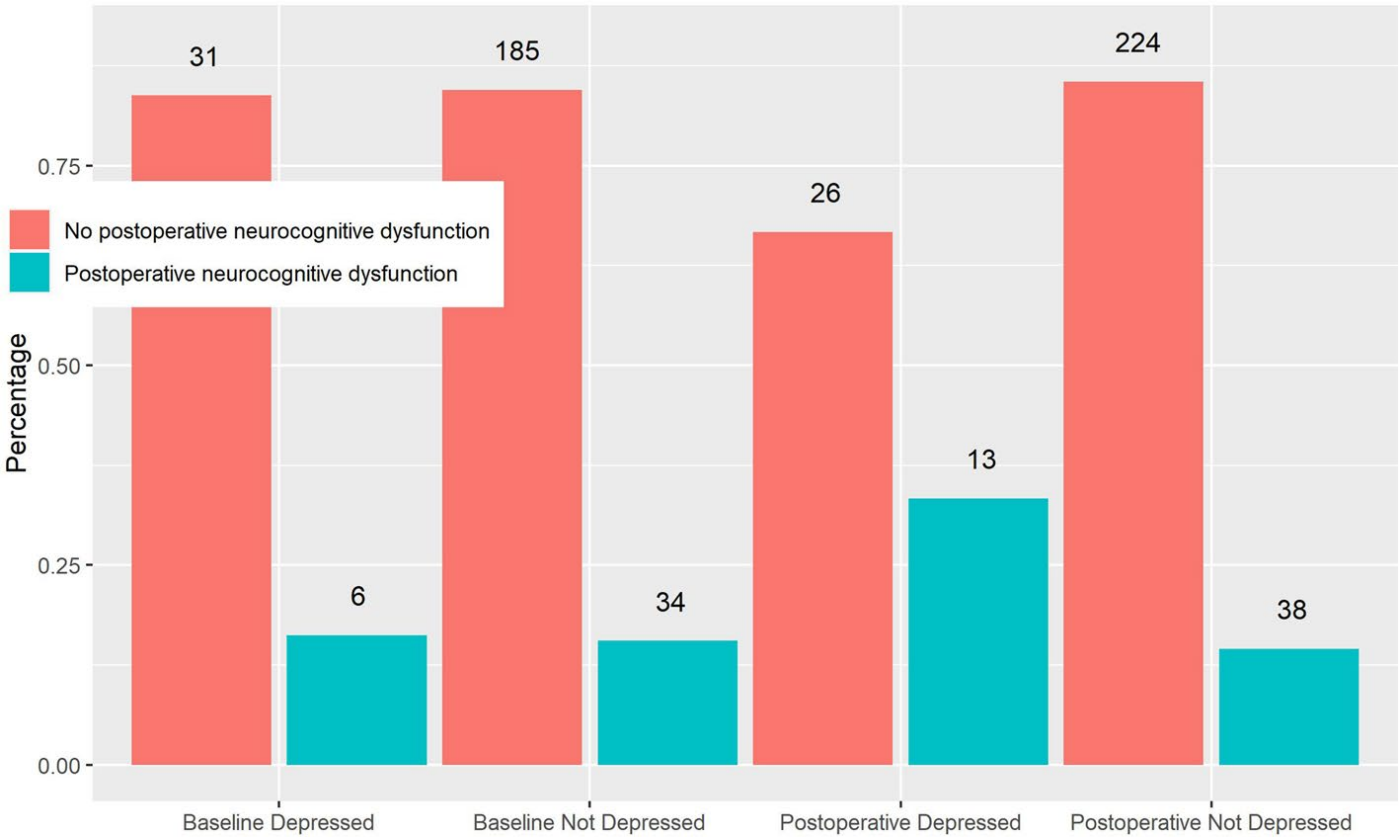
Percentage of patients with postoperative neurocognitive dysfunction one month after surgery based on preoperative or postoperative depression. The number above each column represents the number of patients with or without depression at each respective perioperative testing period. Beck Depression Inventory data were available from 320 patients prior to surgery of which 50 (15.6%) demonstrated depression. After surgery, Beck Depression Inventory data were available from322 patients of which 43 (13.4%) demonstrated depression. Postoperative neurocognitive dysfunction occurred in 16.2% of patients with preoperative depression and 15.5% without preoperative depression (*p* = 0.777). Postoperative neurocognitive dysfunction occurred in 33.3% and 14.5% of patients with and without postoperative depression (*p* = 0.040)

The relationship between depression before surgery and postoperative neuropsychological test results by specific cognitive domain are listed in Table 2. There was no relationship between having evidence of depression before surgery and change in postoperative neuropsychological test results adjusted for sex, age, level of education, preoperative cognitive testing results, parent study randomized blood treatment group, and chronic medical conditions (Table 2). These findings were generally consistent in sensitivity analyses with multiple imputations to account for missing data (supplemental Table 2). In contrast to preoperative depression, there was a significant relationship between being classified as having depression one month after surgery with several domain-specific neuropsychological test results after surgery (Table 3). Patients with depression one month after surgery had significantly poorer performance on tests of attention (*p* = 0.030), verbal fluency (*p* = 0.011), processing speed (*p* = 0.044), and fine motor speed (*p* = 0.030). Additionally, when considered as a composite cognitive outcome, there was no difference in the risk of neurocognitive dysfunction one month after surgery from baseline between patients with and without preoperative depression (Fig. 2). Neurocognitive dysfunction one month after surgery was observed in 16.2% of patients with preoperative depression and 15.5% of patients without preoperative depression (*p* = 0.741). In contrast, there was a significant association between postoperative depression and neurocognitive dysfunction one month after surgery (Fig. 2) occurring in 33.3% of patients with postoperative depression compared to 14.5% of patients without postoperative depression (*p* = 0.025).

**Table 2:** Regression analysis evaluating for any relationship between depression at the time of cardiac surgery and *postoperative* change in domain specific neuropsychological test results. Analysis is adjusted for sex, age, education, baseline cognitive domain z-score, parent study randomized blood pressure management treatment arm, diabetes, obesity, prior stroke, hypertension, atrial fibrillation, and COPD. A negative regression coefficient indicates worse performance for patients with depression compared with those without depression. The “N” represents the number of patients with complete data for the specific domain and covariates included in the model. The results remained consistent in multiple imputation models

Cognitive Domain	N*	No Preoperative Depression	Preoperative Depression	Regression Coefficient^b^	Standard Error	P-Value^a^
		Mean (SD)	Mean (SD)			
Attention	209	0.53 (1.01)	0.67 (1.14)	-0.02	0.19	0.944
Memory	209	0.23 (0.67)	0.23 (0.69)	-0.02	0.13	0.944
Visuoconstruction	200	-0.07 (0.84)	-0.03 (0.96)	0.01	0.17	0.944
Verbal Fluency	200	-0.07 (0.57)	-0.07 (0.60)	0.04	0.12	0.944
Processing Speed	202	0.01 (0.67)	-0.02 (1.22)	-0.15	0.16	0.852
Executive Function	183	-0.10 (0.59)	0.22 (0.60)	0.19	0.13	0.439
Fine Motor Speed	183	0.07 (0.56)	-0.07 (1.19)	-0.29	0.15	0.329

^a^False discovery rate correction for multiple testing applied

^b^Regression coefficient interpreted as adjusted mean difference in change in cognitive domain Z-score between Preoperative Depression and No Preoperative Depression groups

**Table 3:** Associations between postoperative depression and test results for specific cognitive domains one month after surgery adjusted for sex, age, education, baseline cognitive domain z-score, parent study randomized blood pressure management treatment arm, diabetes, obesity, prior stroke, hypertension, atrial fibrillation, and COPD. A negative regression coefficient indicates worse performance for patients with depression compared with those without depression The “N” represents the number of patients with complete data

Cognitive Domain	N*	No Postoperative Depression	Postoperative Depression	Regression Coefficient^b^	Standard Error	*P*-Value^a^
		Mean (SD)	Mean (SD)			
Attention	235	0.67 (1.05)	0.30 (0.85)	-0.44	0.18	0.030
Memory	235	0.41 (0.87)	0.02 (0.99)	-0.25	0.13	0.065
Visuoconstruction	222	0.09 (0.98)	-0.13 (1.35)	-0.02	0.17	0.9897
Verbal Fluency	226	0.14 (1.01)	-0.23 (0.89)	-0.37	0.11	0.011
Processing Speed	225	0.18 (0.91)	-0.41 (1.58)	-0.34	0.15	0.044
Executive Function	203	0.11 (0.99)	-0.04 (0.88)	0.07	0.14	0.751
Fine Motor Speed	200	0.29 (0.60)	-0.21 (1.20)	-0.35	0.13	0.030

^a^False discovery rate correction for multiple testing applied

^b^Regression coefficient interpreted as adjusted mean difference in cognitive domain Z-score between Preoperative Depression and No Preoperative Depression groups

Quality of life measures one month after surgery for patients with and without preoperative depression are shown in Table 4A. Compared with patients without depression, patients with preoperative depression had lower self-ratings of energy/fatigue, emotional well-being, and general perception of their health. Patients with preoperative depression reported higher levels of state and trait anxiety one month after surgery compared with non-depressed patients. Postoperative quality of life measures were adversely affected by depression detected in the one month testing period Table 4B. All but physical health limits were statistically negatively associated with postoperative depression. There was no statistically significant difference in the frequency of clinically detected delirium in patients with and without preoperative depression (16% versus 7.4%, *p* = 0.158). No other significant associations were detected with clinical outcomes (results not shown).

**Table 4:** Association between depression and SF-36 and State Anxiety Inventory results measured one month after surgery. The “N” represents the number of patients with complete data for that domain. The State-Trait Anxiety Inventory S scale represents anxiety associated with an event while the T scale is anxiety level as a personal characteristic

Scale	N	No Depression	Depression	Regression Coefficient^a^	Standard Error	*P*-Value^e^
**A. Preoperative Depression**						
Physical Functioning (median [interquartile range]	187	45.00 [30.00, 65.00]	30.00 [12.50, 48.75]	-10.13	4.78	0.060^b^
Physical Health Limits > 0 (%)	189	51 (25.3)	2 (5.9)	-0.65	0.35	0.093^c^
Emotional Health Limits > 0 (%)	189	165 (81.7)	21 (61.8)	-0.21	0.26	0.411^d^
Energy/Fatigue (median [interquartile range])	189	50.00 [35.00, 60.00]	25.00 [20.00, 35.00]	-12.59	4.44	0.025^b^
Emotional Well Being (median [interquartile range])	189	88.00 [78.00, 96.00]	60.00 [52.00, 70.00]	-9.22	3.56	0.028^b^
Social Functioning (median [interquartile range])	187	62.50 [37.50, 84.38]	43.75 [25.00, 50.00]	-9.41	6.39	0.179^b^
Pain (median [interquartile range])	188	55.00 [45.00, 77.50]	43.75 [22.50, 55.00]	-7.31	5.63	0.218^b^
General Health (median [interquartile range])	185	70.00 [60.00, 80.00]	45.00 [31.25, 55.00]	-14.71	3.76	0.001^b^
State Trait Anxiety State Scale (median [interquartile range])	188	27.00 [21.00, 34.00]	42.00 [35.25, 47.50]	5.73	2.24	0.028^b^
State Trait Anxiety Trait Scale (median [IQR])	181	25.00 [22.00, 32.00]	41.50 [38.00, 47.00]	5.15	2.11	0.032^b^
**B. Postoperative Depression**						
Physical Functioning (median [interquartile range])	189	45.00 [30.00, 65.00]	30.00 [12.50, 48.75]	-19.92	4.34	< 0.001^b^
Physical Health Limits > 0 (%)	190	51 (25.3)	2 (5.9)	-0.5952	0.3476	0.087^c^
Emotional Health Limits > 0 (%)	190	165 (81.7)	21 (61.8)	-0.484	0.24	0.049^d^
Energy/Fatigue (median [interquartile range])	190	50.00 [35.00, 60.00]	25.00 [20.00, 35.00]	-19.10	3.82	< 0.001^b^
Emotional Well Being (median [interquartile range])	190	88.00 [78.00, 96.00]	60.00 [52.00, 70.00]	-21.65	2.83	< 0.001^b^
Social Functioning (median [interquartile range])	188	62.50 [37.50, 84.38]	43.75 [25.00, 50.00]	-16.27	5.67	0.005^b^
Pain (median [interquartile range])	189	55.00 [45.00, 77.50]	43.75 [22.50, 55.00]	-22.65	5.00	< 0.001^b^
General Health (median [interquartile range])	187	70.00 [60.00, 80.00]	45.00 [31.25, 55.00]	-17.90	3.36	< 0.001^b^
State Trait Anxiety State Scale (median [interquartile range])	191	27.00 [21.00, 34.00]	42.00 [35.25, 47.50]	13.74	1.87	< 0.001^b^
State Trait Anxiety Trait Scale (median [interquartile range])	183	25.00 [22.00, 32.00]	41.50 [38.00, 47.00]	12.28	1.71	< 0.001^b^

^a^For continuous outcomes, estimate is the linear regression coefficient for depression; for dichotomized outcomes, estimate is the log odds coefficient for depression;

^b^*P*-value from multivariable linear regression model adjusting for age, sex, education, baseline score, treatment arm, diabetes, obesity, prior stroke, hypertension, atrial fibrillation, and COPD;

^c^*P*-value from logistic regression model, with outcome dichotomized at 0, adjusting for baseline score and treatment arm only due to small cell counts;

^d^*P*-value from logistic regression model, with outcome dichotomized at 0, adjusting for age, sex, education, baseline score, treatment arm, diabetes, obesity, prior stroke, hypertension, atrial fibrillation, and COPD

^e^False discovery rate correction for multiple testing applied

## Discussion

In this exploratory study, we found evidence of depression in 15.6% of patients before surgery and in 13.4% of patients after surgery. Of patients with preoperative depression, 50% remained depressed one month after surgery while 6.6% of patients without preoperative depression demonstrated new evidence of depression after surgery. Preoperative depression was not associated with change from baseline in cognitive performance postoperatively. In contrast, depression detected one month after surgery was associated with worse postoperative cognitive performance in the domains of attention, memory, verbal fluency, processing speed, and fine motor function. In contrast to preoperative depression, those with depression after surgery were more likely to demonstrate the composite neurocognitive dysfunction outcome one month after surgery. Preoperative depression was associated with postoperative impairment in several quality of life domains and with higher anxiety. Depression one month after surgery was associated with impairment on all but one of the quality of life domains and with measures of anxiety.

There is a biologically plausible reason to expect that depression might impair neurocognitive recovery from surgery. Monoamine neurotransmitter imbalance in the central nervous system is one proposed mechanism of depression providing the rationale for treatment with drugs that increase their brain concentrations (e.g., tricyclic antidepressants, selective serotonin reuptake inhibitors, monoamine oxidase inhibitors) [27]. Maladaptive inflammatory responses to various stressors leading to elevation in pro-inflammatory cytokines such as interleukin 1β, interleukin 6, and tumor necrosis factor α is another proposed mechanism of depression [28, 29]. Altered neurotransmission and neuroinflammation are both implicated as putative mechanisms of perioperative neurocognitive disorders supporting the potential for a common underlying mechanism that overlaps with depression [11, 30].

In community-dwelling cohorts cognitive impairment has been shown to accompany depression [31]. Neurocognitive dysfunction is an important source of patient morbidity that can complicate recovery from cardiac surgery [11, 32]. In contrast to non-surgical subjects, there are little data, on whether depression is associated with postoperative neurocognitive dysfunction. In this study we did not find a link between preoperative depression and neurocognitive performance one month after surgery. In an analysis of data collected from 123 patients undergoing CABG surgery enrolled in the Neuropsychiatric Outcomes After Heart Surgery (NOAH) study, preoperative depression defined with the Hamilton Depression Rating Scale was detected in 9.75% of patients [33, 34]. In contrast to our finding, preoperative depression was associated with decline in cognitive performance one month after surgery measured with the clinical dementia rating scale sum of boxes (CDR-SB) score. The CDR-SB inventory is a composite measure of cognitive and functional performance used for dementia rating [34]. Postoperative depression screening results were not reported. Meta-analysis of data from non-surgical cohort studies have demonstrated that deficits in executive function, memory, verbal fluency, and attention are more common in depressed than non-depressed subjects. Not unexpectedly, our data and that from secondary analysis of the NOAH study show that there is overlap in the cognitive domains impaired with depression in patients undergoing cardiac surgery as observed in population-based studies. Our data, though, suggests that depression after surgery might additionally impact processing speed and fine motor speed after surgery that could affect functional outcome. Regardless, mild depressive symptoms can fluctuate and this may explain, in part, the absence of depression after surgery in 50% of patients classified as having preoperative depression. Other patients without depression before surgery could be classified as depressed postoperatively. Limiting depression screening to the preoperative period only, thus, may not detect clinically impactful depression after surgery.

Our findings are in contrast to prior work by our group and that of others showing that pre-existing depression increases the risk for perioperative neurocognitive disorders manifested as delirium after cardiac and non-cardiac surgery [13–15, 35, 36]. In our prior report, we found that a history of depression was associated with a three-fold (95% confidence interval, 1.3 to 7.0, *p* = 0.011) increased risk for delirium after cardiac surgery [13]. In the present study, we objectively determined the presence of depressive symptoms rather than classifying patients as having depression based on medical history. This more sensitive measure identified sub-clinical forms of depression since the majority of depressive symptoms were mild. Regardless, whether perioperative depression contributes to postoperative neurocognitive dysfunction, or vice versa, can not be ascertained from this retrospective analysis. Future prospective investigations are necessary to determine if indeed depression is a potentially modifiable risk for postoperative delirium or neurocognitive dysfunction.

One explanation for our findings of reduced cognitive performance in depressed patients after surgery is that affected patients may show reduced effort during neuropsychological testing which can be rigorous. Meta-analysis from population-based studies, though, show that cognitive impairment is a component of depression and not an epiphenomenon that is secondary to low mood [31]. Our data raises the possibility that targeting treatment of postoperative depression might provide a strategy for improving postoperative neurocognitive function. Treatment with antidepressant drugs alone, while safe in patients with cardiac disease, takes weeks to show benefit and then may have sub-optimal efficacy [37, 38]. Members of our team have previously reported results from a randomized study of 123 patients with DSM-IV criteria for major or minor depression within 1 year of cardiac surgery demonstrating the benefits of cognitive behavioral therapy or stress management counseling compared to usual care [4]. Remission of depression occurred by 3 months of therapy in a greater proportion of patients in the cognitive behavior therapy (71%) and the supportive stress management (57%) groups than in the usual care group (33%, *p* = 0.002). Of note is that that study did not focus on neuropsychological end-points detected in the early months after cardiac surgery. Others have shown benefit from an 8-month telephone delivered collaborative care intervention for improving mental health quality of life, physical function and depressive symptoms after CABG surgery [39]. In that study, patients were screened and enrolled before postoperative hospital discharge using the Physical Health Questionnaire-2. A nurse manager performed active follow-up of patients adhering to evidence-based treatment protocols, patient education about their illness, patient history and preferences for treatments while actively involving their primary care physician and transfer of care to a mental health clinician if the patients did not respond to therapy [39, 40].

Depression at the time of cardiac surgery is known to be associated with postoperative complications, slower physical and emotional recovery, impaired quality of life and mortality [3]. [41–43] Our observation of impairment on some quality of life measures and higher anxiety in depressed versus non-depressed patients, thus, confirms data from other studies. The small number of patients in our study, though, limits the interpretation of the relationship between preoperative depression and risk for major complications. History of overt and covert cerebral ischemic injury as well as narrowing of cerebral arterioles (“small vessel disease”) is known to be associated with depression in the general population [44, 45]. In our study patients with depression had a higher prevalence of known stroke before surgery but there was no indication of any difference in the prevalence of cerebral hemodynamic abnormalities in depressed compared with non-depressed patients. That is, the lower limit of cerebral blood flow autoregulation, a parameter that is higher in patients with prior stroke, was not different between these groups [46]. Further, the frequency of impaired cerebral blood flow autoregulation during cardiopulmonary bypass was not different in depressed and non-depressed patients. In a prior study, we found that patients with brain magnetic resonance imaging evidence of cerebral small arteriolar disease had a higher frequency of impaired cerebral autoregulation during cardiopulmonary bypass [47].

In this study, we did not find a relationship between preoperative depression and clinically detected delirium in contrast to our prior studies [13, 14]. Since many episodes of postoperative delirium are manifest as the hypoactive sub-type, failure to perform a structured delirium assessment such as with the Confusion Assessment Method likely led to our failure to account for all episodes of delirium [48]. Our results are likely confounded by the small number of patients with delirium. Patients in this study were enrolled in a prospectively randomized trial evaluating whether basing mean arterial pressure targets during cardiopulmonary bypass based on individualized cerebral autoregulation data reduces the frequency of neurological complications compared with usual care. In that study, we found that patients in the autoregulation intervention arm had a lower frequency of clinically detected delirium and improved performance on test of memory one month after surgery from baseline. In the current depression study, we adjusted analysis for treatment arm of the parent study minimizing any influence of the autoregulation blood pressure management intervention may have on our observations. Another limitation to this study is missing cognitive data. Missing cognitive data usually involved a small number of test and is often due to the patient’s request to not continue neuropsychological testing which can be challenging especially in those with cognitive impairment. We did not a priori impute missing neuropsychological test scores, however, we did perform imputations in a sensitivity analysis. The latter analysis did not markedly change our findings. Although we did not utilize a formal diagnostic schedule administered by a mental health clinician to validate a psychiatric diagnosis of Major Depressive Disorder, presence of increased depressive symptoms determined by using questionnaires such as the Beck Depression Inventory in non-psychiatric settings have been shown to be clinically significant in improving outcomes and harm reduction [49, 50].

In conclusion, this exploratory analysis of data collected from a randomized study suggests that preoperative depression is largely not associated with perioperative neurocognitive dysfunction but it may impact postoperative quality of life. In contrast, depression detected one month after cardiac surgery may be associated with postoperative impairment in attention, verbal fluency, processing speed, and fine motor function as well as altered quality of life.

## Supplementary Information

**Additional file 1:** **Supplemental Table1. **Patient demographic and operativecharacteristics for patients missing preoperative Beck Depression Inventorydata versus those patients with this data that are included in thisanalysis.

**Additional file 2:** **Supplemental Table 2.**Primary analyses withmultiple imputations evaluating the relationship between preoperativedepression and change in postoperative neurocognitive performance. This analysis was via multivariate imputationby chained equations using 20 imputed datasets.

## Data Availability

Per the charter of the original grant submission, the database from this study will be made available by the corresponding author upon a reasonable request with a clear statement of purpose and aims.
