# Supplementary material for: Postoperative but not preoperative depression is associated with cognitive impairment after cardiac surgery: exploratory analysis of data from a randomized trial
Source: BMC Anesthesiol. 2022 May 23;22:157. doi: 10.1186/s12871-022-01672-y (PMC9125857; doi:10.1186/s12871-022-01672-y)
Supplement: Supplementary file 1 — Additional file 1: Supplemental Table1. Patient demographic and operativecharacteristics for patients missing preoperative Beck Depression Inventorydata versus those patients with this data that are included in thisanalysis. [file 12871_2022_1672_MOESM1_ESM.docx]

**Supplemental Table 1.** Patient demographic and operative characteristics for patients missing preoperative Beck Depression Inventory data versus those patients with this data that are included in this analysis.

|  | **Patients with missing Depression Data not included in this analysis**  **N=140** | **Patients with Beck Depression Inventory Data**  **N=320** | **P-Value^2^** |
| --- | --- | --- | --- |
| Age (mean (SD)) | 70.61 (7.56) | 70.33 (7.71) | 0.714 |
| Gender (%)^1^ | 40 (28.6) | 89 (27.9) | 0.910 |
| Race (%)^1^ |  |  |  |
| White | 112 (80.0) | 261 (81.8) | 0.966 |
| African American | 16 (11.4) | 36 (11.3) |  |
| Asian-Oriental | 1 ( 0.7) | 3 ( 0.9) |  |
| Asian-Subcontinent | 1 ( 0.7) | 3 ( 0.9) |  |
| Hispanic | 2 ( 1.4) | 3 ( 0.9) |  |
| Multiple | 1 ( 0.7) | 1 ( 0.3) |  |
| Other | 7 ( 5.0) | 12 ( 3.8) |  |
| Diabetes (%)^1^ | 72 (52.9) | 137 (43.2) | 0.064 |
| Hypertension (%) | 131 (93.6) | 289 (90.3) | 0.286 |
| Prior stroke (%)^1^ | 15 (11.2) | 29 ( 9.4) | 0.605 |
| Prior myocardial infarction (%)^1^ | 49 (35.8) | 90 (28.3) | 0.121 |
| Atrial fibrillation (%)^1^ | 31 (22.6) | 71 (22.3) | 0.999 |
| Chronic obstructive pulmonary disease (%)^1^ | 13 ( 9.6) | 39 (12.5) | 0.425 |
| Current tobacco use(%)^1^ | 15 (11.0) | 31 ( 9.8) | 0.735 |
| Years of attained education (median [IQR]) | 14.00 [12.00, 17.00] | 14.00 [12.00, 17.00] | 0.979 |
| Anti-depressant drug usage (%) | 19 (13.6) | 43 (13.4) | 0.999 |
| Surgical procedure (%) |  |  | 0.537 |
| CABG | 78 (55.7) | 148 (46.2) |  |
| CABG/AVR | 16 (11.4) | 45 (14.1) |  |
| CABG/MVR | 5 (3.6) | 10 (3.1) |  |
| CABG/AVR/MVR | 23 (16.4) | 66 (20.6) |  |
| AVR | 10 (7.1) | 31 (9.7) |  |
| MVR | 1 (0.7) | 5 (1.6) |  |
| AVR/MVR | 3 (2.1) | 11 (3.4) |  |
| Aortic root replacement | 2 (1.4) | 2 (0.6) |  |
| CABG/Aortic Root Replacement | 1 (0.7) | 0 (0.0) |  |
| TVR | 1 (0.7) | 2 (0.6) |  |
| Minutes of cardiopulmonary bypass (median [interquartile range] ) | 99.00 [78.75, 139.75] | 106.50 [82.00, 140.75] | 0.529 |
| Duration of aortic cross-clamping (median [interquartile range] ) | 66.00 [51.00, 89.00] | 73.00 [55.75, 94.25] | 0.105 |
| Impaired cerebral autoregulation during cardiopulmonary bypass (%) | 48 (34.3) | 82 (26.2) | 0.092 |
| MAP at the lower limit of autoregulation (median [interquartile range] mmHg) | 65.00 [60.00, 75.00] | 70.00 [60.00, 75.00] | 0.427 |
| Area under the curve that MAP was ≤ the lower limit of cerebral autoregulation (median [interquartile range] mmHgxhr)^1.^ | 6.63 [2.13, 14.43] | 6.75 [2.52, 15.10] | 0.804 |
