# Supplementary material for: Postoperative but not preoperative depression is associated with cognitive impairment after cardiac surgery: exploratory analysis of data from a randomized trial
Source: BMC Anesthesiol. 2022 May 23;22:157. doi: 10.1186/s12871-022-01672-y (PMC9125857; doi:10.1186/s12871-022-01672-y)
Supplement: Supplementary file 2 — Additional file 2: Supplemental Table 2.Primary analyses withmultiple imputations evaluating the relationship between preoperativedepression and change in postoperative neurocognitive performance. This analysis was via multivariate imputationby chained equations using 20 imputed datasets. [file 12871_2022_1672_MOESM2_ESM.docx]

**Supplemental Table 2.** Primary analyses with multiple imputations evaluating the relationship between preoperative depression and change in postoperative neurocognitive performance. This analysis was via multivariate imputation by chained equations using 20 imputed datasets.

*Postoperative Neuropsychological Test Results*

| Cognitive Domain | Regression Coefficient | Standard Error | P-value^1^ |
| --- | --- | --- | --- |
| Attention | -0.024 | 0.155 | 0.984 |
| Memory | -0.003 | 0.126 | 0.984 |
| Visuoconstruction | -0.009 | 0.162 | 0.984 |
| VerbalFfluency | 0.047 | 0.108 | 0.984 |
| Processing Speed | -0.065 | 0.136 | 0.984 |
| Executive Function | -0.145 | 0.175 | 0.984 |
| Fine Motor Speed | -0.381 | 0.182 | 0.297 |

^1^ False discovery rate correction for multiple testing applied
